# The Key Role of TNF-TNFR2 Interactions in the Modulation of Allergic Inflammation: A Review

**DOI:** 10.3389/fimmu.2018.02572

**Published:** 2018-11-09

**Authors:** Suhana Ahmad, Nor Azrini Azid, Jennifer C. Boer, JitKang Lim, Xin Chen, Magdalena Plebanski, Rohimah Mohamud

**Affiliations:** ^1^Department of Immunology, School of Medical Sciences, Universiti Sains Malaysia, Kelantan, Malaysia; ^2^Department of Immunology and Pathology, Monash University, Melbourne, VIC, Australia; ^3^School of Chemical Engineering, Universiti Sains Malaysia, Pulau Pinang, Malaysia; ^4^State Key Laboratory of Quality Research in Chinese Medicine, Institute of Chinese Medical Sciences, University of Macau, Taipa, China; ^5^School of Health and Biomedical Sciences, RMIT, Melbourne, VIC, Australia; ^6^Hospital Universiti Sains Malaysia, Universiti Sains Malaysia, Kelantan, Malaysia

**Keywords:** allergy, TNF, TNFR2, regulatory T cells, tolerogenic dendritic cells

## Abstract

Tumor necrosis factor-alpha (TNF) is a pleiotropic cytokine, which is thought to play a major role in the pathogenesis of inflammatory diseases, including allergy. TNF is produced at the early stage of allergen sensitization, and then continues to promote the inflammation cascade in the effector phase of allergic reactions. Consequently, anti-TNF treatment has been proposed as a potential therapeutic option. However, recent studies reveal anti-intuitive effects of TNF in the activation and proliferative expansion of immunosuppressive Tregs, tolerogenic DCs and MDSCs. This immunosuppressive effect of TNF is mediated by TNFR2, which is preferentially expressed by immunosuppressive cells. These findings redefine the role of TNF in allergic reaction, and suggest that targeting TNF-TNFR2 interaction itself may represent a novel strategy in the treatment of allergy.

**Highlights**

- Pleiotropic function of TNF in allergy is likely mediated by its two receptors, TNFR1 and TNFR2
- Activation by TNFR1 results in the allergic inflammatory responses while TNFR2 plays a role in the immune tolerance to allergens
- TNFR2 is preferentially expressed by highly suppressive and replicating Tregs and TNFR2 signaling leads to the activation and proliferation of Tregs
- Targeting of TNFR2 to boost Treg activity may represent a novel strategy for treating patients with allergy.

## Introduction

Allergy is an immune-mediated hypersensitivity to allergens. Exposure of allergens through inhalation, ingestion or skin contact leads to diseases such as asthma, allergic rhinitis, food allergy, and atopic dermatitis. Allergy is a complex disease, and both genetic and environmental factors contribute to its pathogenesis. It affects 30–35% of the population at some point in their life, with incidence continuing to rapidly grow each year. The past decades have exhibited large-scale anthropogenic changes which are currently considered the leading causes of the increasing burden of allergic diseases.

## Effector mechanisms in allergic reactions

**Figure 1:**
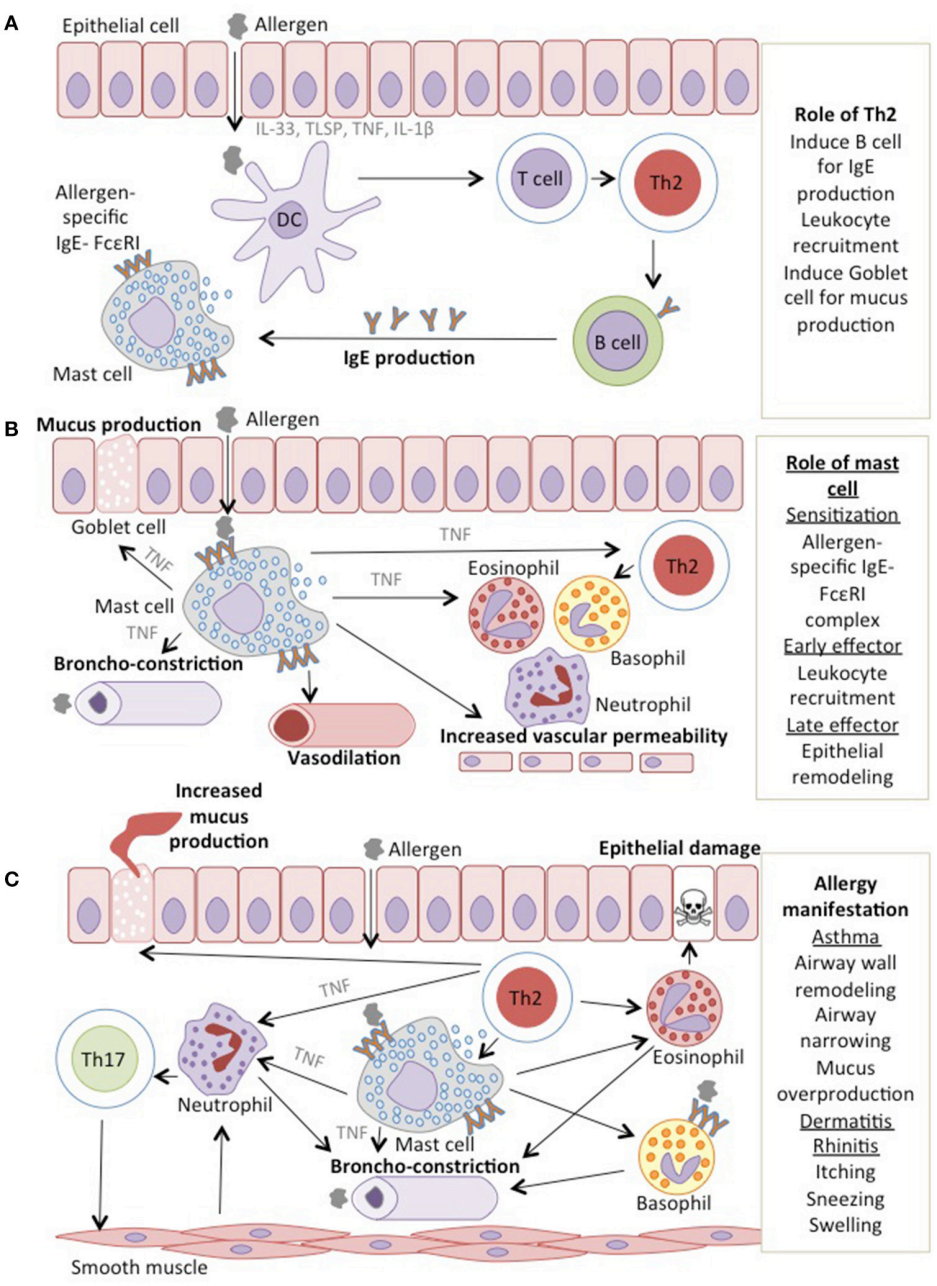
Mechanisms of allergic reactions **(A)**. In sensitization phase, the environmental allergen sensitized epithelial cells and release pro-inflammatory cytokines including TNF. Allergen is taken up by DCs, which are regulated by and produced TNF, induce Th2 cells and drive IgE synthesis and later produced a memory pool of allergen-specific T and B cells **(B)**. Next encounter with the allergen induces assembly with antigen-specific IgE-FcεRI complex on mast cells that secretes TNF and later recruits leukocytes. The leukocytes; basophils, neutrophils, eosinophils and mast cells interact with each other to produce more mucus **(C)**. In the late effector phase, the epithelial remodeling will exhibit the allergic manifestation as in asthmatic and rhinitis patients, the airway wall are narrowed and mucus overproduced while in dermatitis patients, the vasodilation resulted in the itching and swelling of the skin.

The development of allergy can be divided into two phases: (1) the sensitization and memory phase, and (2) the effector phase, which can be further staged into immediate and late responses (1) (Figure 1). Sensitization occurs upon the first encounter with allergen that leads to the production of pro-inflammatory cytokines [Interleukin (IL) 33 (IL-33), thymic stromal lymphopoietin (TSLP), tumor necrosis factor (TNF), IL-1β] by epithelial cells. This allergic sensitization is determined by both genetic polymorphisms (2) and environmental risk factors (3). Studies have identified several risk alleles, including cadherin-related protein 3 (CDHR3) and protocadherin 1 (PCDH1), which are both thought to be involved in facilitating allergic sensitization (4). The ubiquitous presence of lipopolysaccharide (LPS) in the environment may also contribute to the exacerbation of allergic responses. Exposure to LPS triggers signaling of toll-like receptor 4 (TLR4) on epithelial cells and can either promote or suppress the sensitization to an allergen in a dose-dependent manner (5, 6). Allergy is primarily a T helper 2 (Th2)-driven disease (7) in which dendritic cells (DCs) stimulated with cytokines released by sensitized epithelial cells have the capacity to induce Th2 responses. This cascade of events drives IgE synthesis and promotes the generation of memory allergen-specific T and B cells (8).

The effector phase of allergic responses is initiated when the allergen cross-links IgE-FcεRI complexes on sensitized mast cells. Subsequently, mediators of the allergic responses such as histamine, leukotrienes, cytokines, chemokines and proteases, largely responsible for type 1 hypersensitivity are released (9). The release of these mediators causes the acute signs and symptoms of allergy, such as vasodilation and airway constriction. While the continuous exposure to allergen activates T cells and consequently triggers the late phase response, it is the allergen-specific Th2 cells that produce IL-4, IL-5, IL-9, and IL-13, which are responsible for the maintenance of allergen-specific IgE levels and activation of tissue eosinophilia, mucus overproduction, and tissue remodeling (10–13).

Furthermore, Th17 cells were shown to be associated with a more severe asthma, which is less responsive to corticosteroid (14), induces neutrophils recruitment (15) and increases airway inflammation and remodeling (16). In addition to Th1, Th2, and Th17 cells, other cell types are also involved in allergy. For example, Th9 and Th22 cells have been shown to play a crucial role in both early and chronic allergic inflammation. The Th9 cells are vital for the recruitment and activation of mast cells during early allergic response and their increased number in allergic patients is correlated with elevated IgE levels (17). On the other hand, Th22 cells are increased in children with asthma and atopic dermatitis. These cells act by increasing the recruitment of leukocytes and disrupting the epithelial integrity on the skin and in the lungs (18, 19).

Another subset of CD4 T cells, which play an indispensable role in the induction and maintenance of tolerance to allergens, are the immunosuppressive CD4^+^Foxp3^+^ regulatory T cells (Tregs) (20). In one study, the CD4^+^CD25^+^FoxP3^+^ Treg numbers were found to be normal but the expression of FoxP3 protein, a critical marker for Tregs (21) was diminished. However, the authors did not investigate whether there are functional consequences related to a reduced FoxP3 expression in these cells (22). In another study, Fontenot et al., found that FoxP3 displayed reduced expression in patients with allergic rhinitis (23). Because in general, patients with FoxP3 mutations exhibit excessive autoimmunity with high levels of IgE, peripheral eosinophilia and Th2 skewing, the reduced FoxP3 expression may be a contributing factor to the development of allergic diseases in humans (24). Furthermore, in addition to Th2 responses, other types of effector T cells (Teff) are also attributable to the pathogenesis of allergy (25). In the stage of allergy sensitization, Th1 responses are inhibited through reduction of IL-12. However, in the later stage of allergic response, Th1 cells have been shown to coexist with Th2 cells. Such Th1 cells can exacerbate the symptoms and lead to severe allergy manifestation (26).

Overall a proper maturity and homeostasis of immune system of neonates during the first years of life is fundamental to minimize allergic development. Maternal allergy correlates well with impaired frequency and function of Tregs in neonates and consequently the increased susceptibility for the development of allergy in early childhood (27–29). A study found that Th2 cells were increased in newborns' cord blood with maternal allergy, accompanied by a decreased Tregs/Th2 ratio, indicative of an increased risk for developing atopic dermatitis (29). Furthermore, prenatal environmental exposure such as smoking and the usage of harsh chemicals like disinfectants were found to be associated with reduced Tregs number in newborns' cord blood, which further increases the risk of development of allergy later in life (28). Therefore, as shown by these and other extensive studies [reviewed by (30)], Tregs play a fundamental role in the pathogenesis of allergy and their modulation may harness great potential for treatment of allergic diseases.

## General biology of TNF

TNF is a pleiotropic cytokine that plays important dual roles in maintaining immune homeostasis and in promoting the development of diseases. TNF is required for host defense against pathogens, immune surveillance against malignancies, as well as cell proliferation and survival (31). This cytokine is widely considered as an important inflammatory mediator of several diseases such as autoimmune diseases, cancer, hypernociception, cardiovascular disease and fibrosis (32).

Dual biological functions of TNF are likely to be transduced through its two distinct receptors, TNFR1 and TNFR2. TNF and its receptors have both a membrane-bound form and a soluble form. TNFR1 shows high affinity toward both forms of TNF, while TNFR2 is only fully activated by membrane-bound TNF (33). Once activated, TNF elicits its biological functions through the activation of two major signaling pathways, nuclear factor kappa-light-chain-enhancer of activated B cells (NFκB) and mitogen-activated protein kinases (MAPK). These two signaling pathways mediate various cellular activities including proliferation and survival as well as apoptosis and cell death, depending on which receptor is bound and activated by TNF (32).

## Pathogenic role of TNF in allergic manifestation

TNF is reported to play significant role in the pathogenesis of allergy and contributes to both early and late stages of allergy development. This is evident when allergy manifestations are inhibited in TNF-knockout (KO) mice as well as with anti-TNF treatments (34–37). Furthermore, upon allergen exposure, TNF is produced by sensitized epithelial barriers and immune cells (such as macrophages, mast cells, DCs). It has the capacity to promote Th2 responses (38), resulting in high levels of IL-4, IL-5, and IL-13, which further activate eosinophils, mast cells and basophils (7). In allergic rhinitis, TNF is essential for the recruitment of eosinophils to the site of allergic inflammation through the induction of adhesion molecules (34). In asthma, TNF is shown to synergize with IL-17 by promoting neutrophil recruitment (36), whereas in atopic dermatitis, TNF is responsible for the production of other cytokines, including IL-32 which induces keratinocyte apoptosis (39). Furthermore, TNF, both induced and are secreted by Th2 cells which promote the production of antigen-specific IgE isoforms from B cells (34). A meta-analysis study suggested that TNF polymorphisms were significantly associated with asthma susceptibility (40). Elevated TNF levels in severe allergy have been shown to contribute to the epithelial barrier dysfunction by upregulating the adhesion molecules (p120, E-cadherin) and increasing the endothelial permeability to allergens (41).

### Proinflammatory TNF-TNFR1 signaling in allergy

Although binding of TNF to its distinct receptors generally activates the same major signaling pathways (NFκB and MAPK), the distinct structure and motifs of TNFR1 and TNFR2 result in entirely different functional consequences. TNFR1 bears the death domain, which recruits several death signaling proteins such as TNFR1-associated death domain protein (TRADD), Fas associated protein with death domain (FADD) and the TNFR-associated factor (TRAF)-1 to induce inflammation and apoptosis (42).

In the context of apoptosis, TNF-TNFR1 axis signals through caspase 3 and 8, and is mainly responsible for the host defense of pathogen (43) and anti-tumoral activities (44). The impaired regulation of this TNF receptor can cause autoimmune diseases, cancer, chronic infections and allergy (45). In allergic diseases, elevated levels of TNF reportedly trigger inflammatory cascade through TNFR1 (46, 47). For example, Maillet et al. have shown that in a murine model, soluble TNF is a primary driver of allergic airway inflammation, which can be effectively attenuated by neutralization of soluble TNF (48). Increased expression of TNFR1 is shown to play a crucial role in allergic inflammation through the recruitment of eosinophils, neutrophils and other lymphocytes (47). Surprisingly, one study demonstrates anti-apoptotic effects of TNFR1 by enhanced eosinophil survival in asthma, which is contradictory to its known regulation, given the existing cross talk between NFκb and another pathway called c-jun-N-terminal kinase (JNK) pathway (49).

### Immunosuppressive TNF-TNFR2 signaling in allergy

Unlike TNFR1, TNFR2 lacks the death domain where the activation of TNFR2 recruits TNFR-associated factor 2 (TRAF2) that mainly promotes cell proliferation and survival (50). In allergy, impaired TNF-TNFR2 signaling promotes the polarization of Th2 and Th17 cells, thus aggravating the allergy manifestations (51). This notion was further supported by a study showing that TNFR2-KO mice displayed an increased eosinophilic inflammation, one of the most common allergic manifestations, in comparison with wild type mice, whereas TNFR1-KO mice had a weaker response (47). Nevertheless, as TNFR2 promotes cell survival, this receptor signaling also protect inflammatory cells in diseases including eosinophils (52), thus maintain disease progression. Furthermore, TNFR2 signaling on natural killer (NK) cells help to induce Th2 sensitization toward inhaled allergen (53). To add, in certain inflammatory conditions, TNFR2 can also induce apoptosis when it cross-talks with TNFR1 (54). In the cross-talk, TNFR2 induces TRAF2 to deplete cIAP1/2, the apoptosis inhibitor, thus accelerating the TNFR1-dependent apoptosis (54). In addition, with Fas and Fas ligand, TNFR2 signaling induces apoptosis in IFN-γ-Th1 cells, resulting in a predominance of Th2 in atopic individuals (55). Under certain conditions including prolonged cell stress in disease condition, shift of TNFR2 to TNFR1 apoptotic signaling can occur, leading to opposite known function of TNFR2.

**Figure 2:**
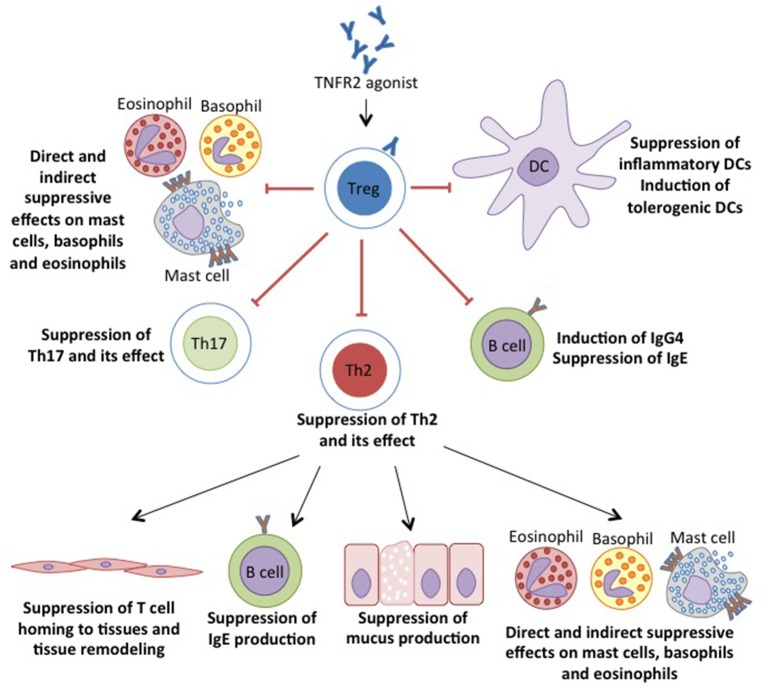
TNFR2 agonist target to enhance the proliferation and suppressive capacity of Tregs. The suppressive Tregs inhibits the inflammation by suppressing the inflammatory cells and induces the tolerogenic DCs by the suppressive MDSCs, hence enabling the suppression of allergic manifestations.

Distinct functions of TNF and its receptor led to the prospective of selectively targeting TNFR1 to inhibit apoptosis, and TNFR2 to induce cell survival. This approach intends to achieve homeostasis in various autoimmune and inflammatory diseases including allergy. These disorders are primarily associated with defects in TNF signaling through TNFR2. Therefore, this particular receptor is of interest and important roles of TNF-TNFR2 interaction on various cell types are further discussed in the next section (Figure 2, Table 1).

**Table 1:** Regulation of TNF on Tregs, DCs and MDSCs for protective immune response against allergic reactions.

**Target cells**	**Protective mechanisms**	**References**
Tregs	Increased suppressive capacity	(56, 57)
	Increased proliferative response	(58, 59)
	Inhibited excessive Th2 and Th17 polarization via inhibition on NF-kB signaling	(51)
DCs	Regulate maturation and survival	(60, 61)
	Tolerogenic DCs	(62, 63, 64)
MDSCs	Inhibit activation of Th2 cells	(65, 66)

#### TNF-TNFR2 on tregs

Due to its important role in allergy manifestations, TNF has been evaluated as a target for therapy and findings have led to the discovery of a more prominent role of TNFR2 in the pathogenesis of allergy. Only a few effects of the exclusive TNF signaling via TNFR2 have been characterized, since its expression is limited to certain cell populations and is only fully activated by membrane-bound TNF. In T cell biology, TNFR2 is directly associated with proliferation and maintenance of function, both in Tregs (67, 68) and Teff (69, 70). What is more interesting is the restricted abundance of TNFR2 on Tregs, leading to strong activity of this receptor on the mediator of tolerance.

TNFR2, which is preferentially expressed on human and mouse Tregs, is associated with both phenotypic and functional properties of Tregs (67). Tregs are shown to inhibit inflammatory responses by TNF through TNFR2 signaling (71). In addition, in diseases, downregulation of Foxp3 has been associated with TNF- TNFR2 interaction and is later restored by blockade of TNF with TNF antagonist (59, 72, 73). Unlike Foxp3 of which the forced expression can convert CD4^+^CD25^−^ Teff into functional Tregs (74), TNFR2, does not induce CD4^+^Foxp3^−^ T cells to become suppressive (70). Instead, TNFR2 expressing CD4^+^Foxp3^−^ T cells show greater resistance to suppression by TNFR2^−^ Tregs. In addition, TNFR2 is also shown to be responsible for a more potent suppressive capacity of Tregs when TNFR2^+^ Tregs preferentially accumulated intratumorally than in periphery (68). Previously, Tregs were described as CD4^+^ T cells expressing CD25, the IL-2R α chain, and CD45RB with Foxp3 as functional transcription marker (75). Co-expression of TNFR2 with CD25 has been suggested to identify more functional suppressive Foxp3^+^ Tregs in human (76). Although TNFR2 induces proliferation of both Tregs and Teff, TNFR2^+^ Tregs are able to overcome the inhibition of suppression of TNFR2^+^ Teff (70). A reduction of Tregs in TNFR2 deficient mice indicates the role of TNFR2 in promoting the generation and homeostasis of this cell subtype (56). Both *in vivo* and *ex vivo* expansions of Tregs while maintaining their suppressive capacity have been demonstrated by utilizing TNFR2 signaling (77, 78).

Furthermore, a subset of Treg, CD8^+^ Tregs, which can be rapidly generated in the presence of IL-4 and IL-12, can both block activation of naïve or Teff and suppress IgG/IgE antibody response (79). Expansion of this Treg subset is induced in the presence of activated CD8^+^ T cells via TNFR2 signaling in which TNFR2 is usually identified on a more potent subpopulation (80, 81). However, there are studies that experimentally demonstrate the inhibition of Tregs suppressive function through TNF-TNFR2 axis (57, 73, 82). These inhibitory effects of TNF-TNFR2 axis in Tregs have been associated with several mechanisms including activation of NFκB cascade, preferentially activated by pro-inflammatory TNFR1, (82) and activation of Akt with Smad3 that reduced Foxp3 transcription (57). This discrepancy of TNFR2 signaling on the function of Tregs is hypothesized to be related with the crosstalk of TNFR2 with TNFR1. Under certain conditions including prolonged cell stress in disease condition, shift of TNFR2 to TNFR1 apoptotic signaling can occur, leading to opposite primary function of TNFR2 (50).

#### TNF-TNFR2 on DCs

The biological function of DCs, the professional APCs, can be regulated by TNF. Conversely, TNF is produced by DCs upon exposure to exogenous antigen or allergen. Subsequently, depending on the cytokine milieu including TNF, DCs would activate distinct T cell responses, which in allergy corresponds primarily to a Th2 response (83). Furthermore, TNF differentially regulates maturation and survival of DCs through interaction with its two receptors, TNFR1 and TNFR2. (60). Interaction between TNFR2 and membrane-bound TNF expressed by tolerogenic DCs induces generation of suppressive Tregs (84). In addition, TNFR2 also regulates survival of DCs, which is crucial in maintaining immunity (60, 61). These studies showed that TNFR2-deficient mice failed to develop matured myeloid cells and DCs thus reducing T cell activation including Tregs. However, the maturation state of DCs when regulating the immune tolerance has been subject of debates (85). Previously, tolerance in terms of T cell anergy and deletion is induced by immature DCs (86). Currently, mature DCs which are characterized by the typical phenotypic expression of CD103, are capable of promoting antigen specific Tregs (87). CD103^+^ DCs have been shown to selectively prime Th2 responses to inhaled allergens and impaired allergic airway inflammation in mice lacking CD103^+^ DCs (88). Alternately, CD103^+^ DCs in lymphoid organs have the capacity to promote generation of Foxp3^+^ Tregs by metabolizing dietary vitamin A (89). Also, CD103^+^ DCs in the airway has been shown to restrain airway inflammation, for example through the induction of IL-10 (62) and production of IL-12 that control Th1 (90). Previously, it was shown that TNFR2 maintains adequate IL-12 production by DCs in inflammatory responses by regulating endogenous TNF level (91). In addition, selective expansion of maximally suppressive TNFR2^+^Foxp3^+^ Tregs in pulmonary inflammation can be induced by engineered nanoparticles through the activation of CD103^+^ DCs (92).

#### TNF-TNFR2 on MDSCs

Furthermore, TNFR2 also acts as a co-stimulatory receptor, crucial for the development and regulation of myeloid suppressor cells (61). Myeloid-derived suppressor cells (MDSCs) are an innate heterogeneous cell population that plays a crucial role in dampening inflammatory responses. The accumulation and expansion of MDSCs has been observed in several diseases like tumors, infectious diseases, trauma, autoimmune diseases and asthma as well. The suppressive MDSCs regulate the adaptive immunity by inhibiting the activation of T cells, especially Th2 cells in allergy (65, 66), although the upregulation of MDSCs, synergistically with Th2 polarization in asthma has also been observed (65). Similar to Tregs, the TNF-TNFR2 axis also promotes the activation and suppression of MDSCs in tumor progression by several mechanisms such as promoting secretion of nitric oxide (NO), IL-10 and TGF-β as well as enhancing the inhibition of lymphocyte proliferation (93, 94). However, it is unclear whether the interaction of TNFR2 with Tregs and MDSCs have the same signaling pathway of the suppressive mechanism in tumor development and immune evasion. For example the activation of TNFR2 on Tregs enhances immune suppression *in vivo* and stimulates proliferation *in vitro* (61) while MDSCs rely on TNFR2 activation for their maturation and to reach their optimal suppressive function (95).

## Therapeutic targeting of TNF-TNFR2 in allergy

Currently, treatment for allergy such as antihistamine and glucocorticoid only temporarily relieve symptoms in terms of inflammation, hence a disease-modifying treatment is fundamental. In allergic models, TNF antagonist have been shown to induce anti-allergic effects by reducing IgE level, Th2 cytokines, and eosinophils infiltration (35, 48, 96). Moreover, preferential expression of TNFR2 on Tregs makes it a more attractive molecular target for drug development. Studies showing the efficacy of targeting TNF-TNFR2 in allergies such as atopic dermatitis (97) and asthma (46, 59) have made this as a promising treatment option. Although such molecules are capable of neutralizing TNF, their affinity and avidity toward both soluble and transmembrane TNF is highly variable as well as the effects they exert on TNF-producing cells. The big differences can specifically be found in their distinct pharmalogical properties, which explains their variable efficacy in allergy treatment modalities (98) (Table 2).

**Table 2:** TNF antagonist and its efficacy in allergy as well as their effects on Tregs.

**Biologic name (trade name)**	**Type of agent**	**Approved indication**	**Efficacy in allergy**		**Effects on Tregs**	**References**
			***in vitro***	***in vivo***	
Etanercept (Enbrel)	Human TNFR2-Fc fusion protein	RA, JIA, PA, AS, PP	+++	+	Decrease of Foxp3+ cells *in vitro*.	(66, 96, 97, 98, 99, 100)
Infliximab (Remicade)	Chimeric anti-TNF mAb	RA, CD, UC, AS, PS, PP	+++	+	Expansion of Tregs in RA	(34, 70, 93, 94, 101)
Adalimumab (Humira)	Human anti-TNF mAb	RA, PA, CD, PP	nd	Nd	Expansion of Tregs in RA	(99, 102)
Golimumab (Simponi)	Human anti-TNF mAb	RA, AS, PA	nd	–	nd	(103, 104)
Certolizumab Pegol (Cimzia)	Human PEGylated Fab anti-TNF mAb	RA, CD, PA, AS	nd	nd	nd	(105)

*RA, rheumatoid arthritis; AS, ankylosing spondylitis; CD, Crohn's disease; PP, plaque psoriasis; PA, psoriatic arthritis; UC, ulcerative colitis; JIA, juvenile idiopathic arthritis; +++, very strong; +, weak; -, no effect; nd; no data*.

In clinical settings, five TNF antagonists (three human monoclonal antibodies; infliximab, adalimumab, and golimumab, a TNFR2 receptor etanercept and certolizumab, the PEGylated Fab antibodies) have been established as therapeutic options in inflammatory diseases. Etanercept, a genetically engineered recombinant protein that comprises of TNFR2 and Fc portion of human IgG1, specifically binds to TNF and blocks its interaction with cell surface receptors (99). Studies have shown that in an allergy model, etanercept attenuates allergic lung inflammation (48) while in allergic asthma it can even reverse the inhibitory activity of TNF onto Tregs (59). However, in a randomized, phase II controlled trial (RCT), in comparison to placebo, etanercept only showed to be a well-tolerated therapy in moderate-to-severe asthma but with no significant clinical efficacy (100). Targeting TNF with etanercept in mild-to-moderate allergic asthma increased the TNFR2 levels but failed to attenuate disease pathologies (101). Moreover, treatment with etanercept in severe atopic dermatitis only showed modest effects (102). Infliximab, the chimeric monoclonal anti-TNF, showed significantly, reduced pathological inflammation in allergy model (35, 96). In addition, treatment with infliximab improved clinical outcome in both moderate and severe atopic dermatitis, but failed to respond in the maintenance therapy (97). Another anti-TNF monoclonal antibodies, golimumab, demonstrated to be unsuitable for treatment in severe asthma when it shows unfavorable risk-benefit profile in a RCT (103). Although it is well established that TNF plays a prominent role in establishment and maintenance of allergy, treatment with its antagonist in allergy population shows to be inefficient although they successfully attenuate the symptoms in allergy model. The severe side effects (immunosuppression, risk of infection, hematological malignancies, demyelinating events and neuropathies, impact on cardiovascular), and unsuccessful trials, are a limitation to their use as a general treatment in allergy (42).

Unlike in allergy, TNF-TNFR2 axis in autoimmune diseases and cancer has been well established. Adalimumab has shown to expand functional Tregs through TNF-TNFR2 axis on monocytes in rheumatoid arthritis (104). Another study in a rheumatoid arthritis model exhibited selective blockade of TNFR1 while sparing TNFR2 ameliorated inflammation and enhanced number and function of Tregs (105). Nguyen and Ehrenstein (104) have demonstrated a mechanism where TNF antagonism selectively neutralized the pro-inflammatory soluble TNF while it enhanced the immunosuppressive function of membrane TNF (104). The aforementioned studies may suggest that targeting TNF through TNFR2 not only neutralizes its pro-inflammatory activities but also induces tolerance by activation and expansion of Tregs (Table 2). In allergy perspective, although TNF antagonism does increase the TNFR2 levels (101), but other study demonstrated a functional insufficiency by TNF via TNFR2 signaling pathway (59). Interestingly, effectiveness toward anti-TNF treatment has also been associated with polymorphism of TNF receptor superfamily member 1B that code for TNFR2 protein (106). This may explain the effectiveness of TNF antagonism in only certain allergy population.

Due to its restricted cellular expression and prominent role in immune regulation, TNFR2 is still a more attractive target for treatment in diseases, including allergy. Faustman and Davis (50) suggested both short and long-term strategies to refine TNFR2 as a target in therapy. These include a better TNFR2 agonist by mean to improve specificity, binding duration, and affinity, as well as TNF inducers, modulation in NF-kB pathway and CD3-specific antibodies (107). Furthermore, utilization of nanoparticles to modulate immune response has been widely investigated (108). Nanoparticles, with their various immunological effects in the lung (109), can be utilized to selectively block TNFR1 and/or activate TNFR2. A synthetic nanoparticle has been used to imprint innate immunity when pre-exposed mice preferentially expanded TNFR2^+^Foxp3^+^ Tregs after allergen challenge, partly via the activation of CD103^+^ DCs (92, 110). This non-toxic engineered nanoparticle evidenced the selective modulation of Tregs homeostasis through mechanisms such as maintenance of TNF-TNFR2 interaction, targeting CD103^+^ DCs, and expanding the proliferative rate of Tregs, thus decreasing the susceptibility to allergic disease. This strategy to use engineered targeting can also be adopted in specific immunotherapy (SIT). It can be considered as an achievable long-term cure for allergy as basic principle in SIT, which involves inducing immune tolerance toward allergens by specifically and repeatedly administrating the causative allergen (111).

## Conclusive remarks

In allergy, TNF may have dual pro-inflammatory and anti-inflammatory activities, which are likely mediated by its two distinct receptors. TNFR2 signaling is attributable to the immunosuppressive effects of TNF and thus is protective against allergy. Consequently, targeting TNF-TNFR2 pathway may represent future direction to develop new therapies in allergy. However, as a pleiotropic cytokine, TNF and its effects on TNFR signaling is diverse and can either regulate allergic manifestations or to control disease pathogenesis. Implications of TNFR2 in health and diseases requires additional investigation to further elucidate their exact mechanisms and provide more insight for future strategies in manipulating TNFR2 for therapeutics.

## Author contributions

All authors listed have made a substantial, direct and intellectual contribution to the work, and approved it for publication.

### Conflict of interest statement

The authors declare that the research was conducted in the absence of any commercial or financial relationships that could be construed as a potential conflict of interest.

## Abbreviations

CDHR3: Cadherin-related protein 3
DCs: Dendritic cells
Interleukin: IL
IL-17RD: Interleukin 17 receptor D
MAPK: Mitogen-activated protein kinases
MDSC: Myeloid-derived suppressor cell
NFκB: Nuclear factor kappa-light-chain-enhancer of activated B cells
NK: Natural killer cells
NO: nitric oxide
PCDH1: Protocadherin 1
RCT: Randomized controlled trial
SIT: Specific immunotherapy
Teff: Effector T cells
Th: T helper cells
TNF: Tumor necrosis factor
TNFR: Tumor necrosis factor receptor
TRADD: TNFR-associated death domain protein
TRAF: TNFR-associated factor
Tregs: Regulatory T cells
TSLP: Thymic stromal lymphopoietin.
